# A *GJA9* frameshift variant is associated with polyneuropathy in Leonberger dogs

**DOI:** 10.1186/s12864-017-4081-z

**Published:** 2017-08-25

**Authors:** Doreen Becker, Katie M. Minor, Anna Letko, Kari J. Ekenstedt, Vidhya Jagannathan, Tosso Leeb, G. Diane Shelton, James R. Mickelson, Cord Drögemüller

**Affiliations:** 1 Institute of Genetics, Vetsuisse Faculty, University of Bern, 3001 Bern, Switzerland; 2 Department of Veterinary and Biomedical Sciences, College of Veterinary Medicine, University of Minnesota, St. Paul, MN 55108 USA; 3 Department of Basic Medical Sciences, College of Veterinary Medicine, Purdue University, West Lafayette, Indiana, 47907 USA; 4 Department of Pathology, School of Medicine, University of California, San Diego, La Jolla, California, 92093 USA

**Keywords:** Dog, Rare disease, Neurological disorder, Peripheral nerve, Polyneuropathy, Charcot-Marie-tooth, Genome wide association, Whole-genome resequencing, Gene test, Connexons, Connexin genes, Gap junctions

## Abstract

**Background:**

Many inherited polyneuropathies (PN) observed in dogs have clinical similarities to the genetically heterogeneous group of Charcot-Marie-Tooth (CMT) peripheral neuropathies in humans. The canine disorders collectively show a variable expression of progressive clinical signs and ages of onset, and different breed prevalences. Previously in the Leonberger breed, a variant highly associated with a juvenile-onset PN was identified in the canine orthologue of a CMT-associated gene. As this deletion in *ARHGEF10* (termed LPN1) does not explain all cases, PN in this breed may encompass variants in several genes with similar clinical and histopathological features.

**Results:**

A genome-wide comparison of 173 k SNP genotypes of 176 cases, excluding dogs homozygous for the *ARHGEF10* variant, and 138 controls, was carried out to detect further PN-associated variants. A single suggestive significant association signal on CFA15 was found. The genome of a PN-affected Leonberger homozygous for the associated haplotype was sequenced and variants in the 7.7 Mb sized critical interval were identified. These variants were filtered against a database of variants observed in 202 genomes of various dog breeds and 3 wolves, and 6 private variants in protein-coding genes, all in complete linkage disequilibrium, plus 92 non-coding variants were revealed. Five of the coding variants were predicted to have low or moderate effect on the encoded protein, whereas a 2 bp deletion in *GJA9* results in a frameshift of high impact. *GJA9* encodes connexin 59, a connexin gap junction family protein, and belongs to a group of CMT-associated genes that have emerged as important components of peripheral myelinated nerve fibers. The association between the *GJA9* variant and PN was confirmed in an independent cohort of 296 cases and 312 controls. Population studies showed a dominant mode of inheritance, an average age of onset of approximately 6 years, and incomplete penetrance.

**Conclusions:**

This *GJA9* variant represents a highly probable candidate variant for another form of PN in Leonberger dogs, which we have designated LPN2, and a new candidate gene for CMT disease. To date, approximately every third PN-diagnosed Leonberger dog can be explained by the *ARHGEF10* or *GJA9* variants, and we assume that additional genetic heterogeneity in this condition exists in the breed.

**Electronic supplementary material:**

The online version of this article (10.1186/s12864-017-4081-z) contains supplementary material, which is available to authorized users.

## Background

Several studies have reported the occurrence of peripheral neuropathies (PN) in many breeds of dogs [1–4]. Affected dogs present with abnormal motor signs including weakness, hypotonia and muscle atrophy secondary to denervation [1]. Focal signs such as laryngeal paralysis, a change in the pitch of the bark, inspiratory stridor, and dyspnea, can be the only clinical abnormalities early in the progressive course of the disease and are considered as strong indicators of the presence of an underlying neuropathy that may later progress [1–3]. An observational study across breeds revealed that approximately every second dog with laryngeal paralysis had evidence of diffuse PN [3].

The histopathological phenotype seen in many canine PNs is similar to the most common group of inherited polyneuropathies in humans, known as Charcot-Marie-Tooth (CMT) disease [5]. Nearly 30 years ago it was already speculated that some forms of canine PN represent inherited diseases [2]. This was recently confirmed by unraveling causative recessive variants in the canine orthologue of a human CMT-associated gene (*NDRG1*) in Greyhounds [6] and Alaskan Malamute dogs [7] with early-onset PN (OMIA 001292–9615). More recently, *RAB3GAP1*-associated forms of syndromic PN accompanied by ocular abnormalities and neuronal vacuolation (OMIA 001970–9615) were described independently in Black Russian Terriers [8], Rottweilers [9] and Alaskan Husky dogs [10].

The Leonberger breed was formed by the crossing of several large-bodied breeds, including Saint Bernards, Newfoundlands, and Great Pyrenees. Chronic nerve fiber loss associated with axonal degeneration, decreased myelinated fiber density and a shift of the axonal size-frequency distribution toward smaller fibers is the predominant finding in peripheral nerves of PN-affected Leonberger dogs [1, 5]. PN in Leonbergers is twice as frequent in males as females, has a wide age range of onset, and due to the absence of pain and gradual onset of clinical signs, may go undetected for a number of months to years. An *ARHGEF10* loss-of-function 10 bp deletion, affecting another human CMT-associated candidate gene, was shown to be highly associated with a severe juvenile-onset form of PN in Leonberger, as well as Saint Bernard dogs (OMIA 001917–9615) [11]. In a cohort of more than 200 affected Leonberger dogs approximately 80% of all PN cases were found to be negative for the mutant *ARHGEF10* allele [11], suggesting that PN in this breed may be a result of several genetically distinct variants having similar clinical and histopathological features. The goal of the present study was to identify additional disease-associated variants for PN in Leonberger dogs using a positional cloning strategy.

## Methods

### Animals

Genomic DNA was isolated from blood using either the Gentra PureGene blood kit (Qiagen) or the Maxwell RSC whole blood DNA kit (Promega). All dogs were genotyped for an *ARHGEF10* deletion (i.e., the LPN1 allele) as previously described [11], and only dogs that were homozygous wild type or heterozygous for the *ARHGEF10* allele were selected for genotyping on the SNP arrays. The phenotypic characterization of PN in Leonberger dogs has been described elsewhere [5] and the previously established criteria to select cases and controls were applied [11]. Samples from a total of 7455 Leonbergers, including 922 dogs with detailed phenotype records (Additional file 1) were used during this study. Initially, a cohort of 314 dogs was used for the GWAS; later a follow-up cohort of 608 dogs with detailed phenotype information was collected, in addition to 6533 dogs that were submitted without known phenotype status. A total of 56 PN-affected Leonberger dogs homozygous for the deletion in *ARHGEF10* were excluded from the mapping studies.

### Histopathology

As part of a larger study, diagnostic peroneal nerve biopsies collected under general anesthesia or peroneal nerve samples collected at necropsy were immersed into 10% neutral buffered formalin, resin embedded and cut into thick (1 μm) sections for evaluation by a single pathologist (GDS).

### Genome analyses

A total of 314 dogs (176 cases and 138 controls; Additional file 1), were genotyped on the Illumina CanineHD BeadChip (Illumina, San Diego, CA, USA) that contains 173,677 SNP markers. PLINK software [12] was used to prune the genotyping data: (1) to remove SNPs with more than 10% missing genotype calls per marker; (2) to exclude uninformative SNPs with a minor allele frequency below 5%; and (3) to exclude SNPs which exceed the Hardy-Weinberg disequilibrium *p*-value of 0.0001. Population stratification, resulting from close familial relationships, was confirmed by the genomic inflation factor of 1.55 calculated during an association test. Therefore, a mixed model approach utilizing the GenABEL package [13] was applied for the association analysis to correct for this population stratification and any cryptic relatedness. This correction resulted in a genomic inflation factor of 1.04. Haplotypes around the significantly associated locus were constructed using fastPHASE [14]. All genome positions refer to the CanFam3.1 reference sequence assembly.

### Whole genome sequencing and variant calling

Whole genome sequence data at 12× coverage was obtained from a PN-affected Leonberger dog after preparation of a fragment library with a 250 bp insert size and collection of 293,912,640 paired-end reads (2 × 100 bp) using a HiSeq2500 instrument (Illumina). The sequence data analysis and variant calling was performed as described before [15]. The annotation version CanFam3.1.75 (http://www.ensembl.org) was used to predict the functional effects of detected variants as described previously [15]. The genome sequencing data were deposited in the European Nucleotide Archive (ENA, http://www.ebi.ac.uk/ena) under sample accession number SAMEA47266168 within study accession PRJEB16012. The sequence data from the Leonberger case were compared to the Boxer reference genome (CanFam3) and 202 publically available control dogs of 66 various different breeds (Airedale Terrier, Alaskan Husky, Alaskan Malamute, Alpine Dachsbracke, American Staffordshire Terrier, Australian Cattle dog, Australian Shepherd, Australian Terrier, Basenji, Basset, Bavarian Hound, Beagle, Bearded Collie, Berger Blanc Suisse, Border Collie, Bull Terrier, Bullmastiff, Cairn Terrier, Cavalier King Charles Spaniel, Chihuahua, Chinese indigenous dog, Chow Chow, Cocker Spaniel, Curly Coated Retriever, Dachshund, Doberman Pinscher, Elo, Entlebucher Sennenhund, Eurasier, French Bulldog, German Shepherd, German Spitz, German Wirehaired Pointer, Golden Retriever, Great Dane, Greater Swiss Mountain Dog, Greyhound, Heideterrier, Hovawart, Irish Terrier, Jack Russell Terrier, Jagdterrier, Kromfohrländer, Kunming Dog, Labrador Retriever, Lagotto Romagnolo, Landseer, Leonberger, Malinois, Miniature Bull Terrier, Norwich Terrier, Old English Sheepdog, Perro de Agua Español, Pomeranian, Poodle, Rhodesian Ridgeback, Rottweiler, Saluki, Shetland Sheepdog, Siberian Husky, Sloughi, Tibetan Mastiff, Weimaraner, West Highland White Terrier, Whippet, and Yorkshire Terrier) and 3 wolves; deposited in the European Nucleotide Archive (http://www.ebi.ac.uk/ena) under study accession numbers PRJEB10823, PRJEB13139, PRJEB13468, PRJEB13723, PRJEB14110, PRJEB14840, PRJEB16012, PRJEB4544, PRJEB5500, PRJEB5874, PRJEB5875, PRJEB6076, PRJEB6079, PRJEB7734, PRJEB7735, PRJEB7736, PRJEB7903, PRJEB9437, PRJEB9590, PRJEB9591, and PRJNA266585.

### Additional genotyping

Sanger sequencing was used to confirm the Illumina sequencing results and to perform targeted genotyping for 6 variants identified from whole genome sequencing. For these experiments we amplified PCR products using AmpliTaqGold360Mastermix (Life Technologies) and purified PCR products were directly sequenced on an ABI3730 capillary sequencer (Life Technologies). The sequence data were analyzed using Sequencher 5.1 software (GeneCodes).

The *GJA9* deletion was genotyped by fragment size analysis (primers: CCTGACAACCACAGTGGAAA (forward) and AGAGCAGTGGTTCCTTTTGC (reverse)) on an ABI3730 capillary sequencer and analyzed with the GeneMapper 4.0 software (Life Technologies).

Comparison of *GJA9* allele and genotype frequencies in PN cases and controls was performed in the original GWAS cohort, as well as an independent cohort, by standard chi-square tests.

## Results

### GWAS mapping of a PN associated locus on CFA15

**Fig. 1:**
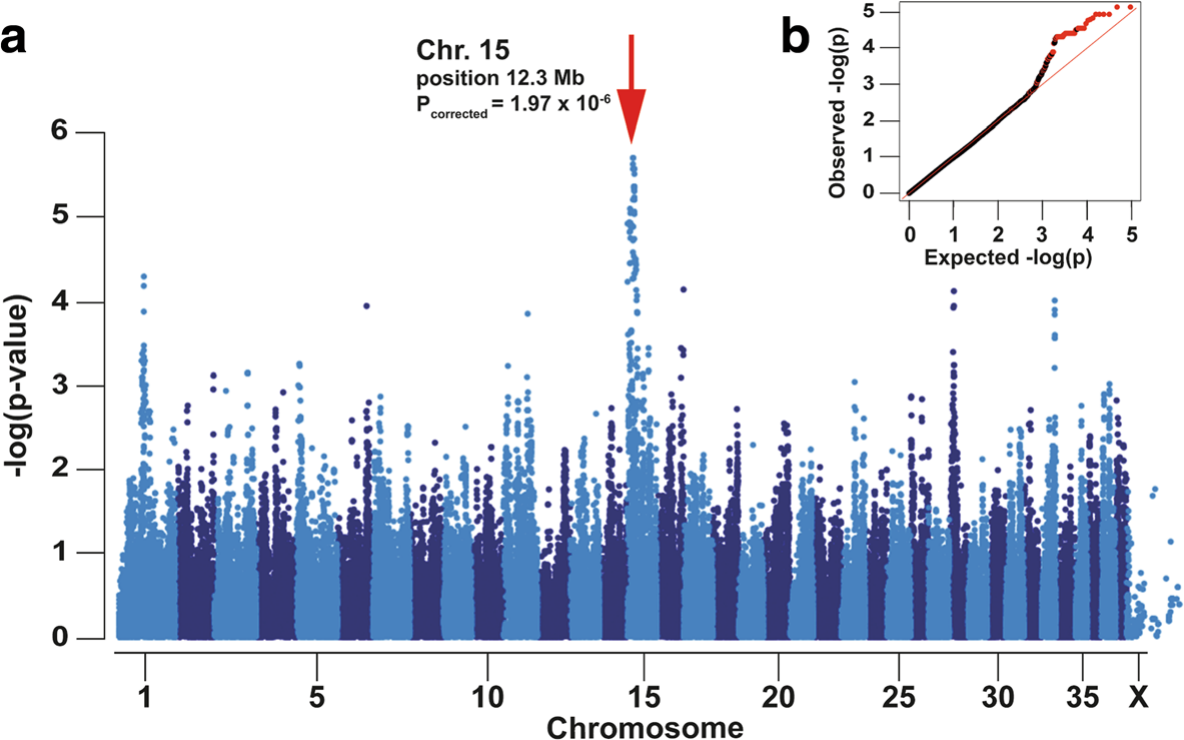
Genome-wide association study for polyneuropathy in Leonberger dogs. **a** Note the association signal on chromosome 15. **b** The quantile–quantile (QQ) plot shows the observed vs. expected log *P*-values. The straight line in the QQ plot indicates the distribution of SNP markers under the null hypothesis, and the skew at the right edge indicates those markers that are more strongly associated with the trait than would be expected by chance. Markers from chromosome 15 are shown in red

After pruning for low genotyping rate, low minor allele frequency, and failure to meet Hardy-Weinberg equilibrium, 108,801 SNPs remained for the association analysis. A suggestive significant association signal on CFA15 was identified, with the two most significant SNPs (BICF2G630442965, at bp position 12,345,641; and BICF2G630442606 at bp position 12,765,329) achieving a corrected *p*-value of 1.97 × 10^−6^ (Fig. 1a; Additional file 2). The quantile-quantile plot of observed versus expected *p*-values of this mixed model analysis also supports the effectiveness of the correction for population structure and the significance of the CFA15 locus (Fig. 1b).

Haplotype reconstruction revealed evidence for an approximately 23 Mb sized disease-associated haplotype at the centromeric end of acrocentric CFA15 (Additional file 3). Five PN-affected dogs were homozygous for this haplotype spanning from bp position 0 to 22,723,949 and 37 other cases were identified as heterozygous carriers of a single copy of the identical 22.7 Mb sized haplotype. Further, 8 PN-affected dogs carried shorter versions of this haplotype due to recombination events, which enabled narrowing of the critical region to approximately 7.7 Mb (bp position 1,762,076 to 9,441,628) (Additional file 3). This region contains 135 annotated protein-encoding genes and 72 other loci.

### Whole genome sequence analysis identifies a frameshift variant in the GJA9 gene

Whole genome sequence was generated from a PN-affected Leonberger homozygous for the associated CFA15 haplotype and variants in the critical interval were identified. We hypothesized that the causative variant should be absent from breeds unrelated to the Leonbergers, as pedigree analysis and the large size of the associated haplotype clearly indicated a relatively young origin of the mutation. A total of 98 variants in the interval unique to the sequenced case remained after filtering against 202 control genomes of 66 different dog breeds and 3 wolves (Additional file 4). This represented 92 homozygous private non-coding variants as well as 6 coding variants, namely: synonymous variants in *AGO3*, *CSMD2* and *GRIK3;* missense variants in *MYCL* and *MACF1* that correspond to the reference alleles in other species, and a 2 bp deletion causing a frameshift in *GJA9* (Additional file 4). All 6 coding variants were genotyped by Sanger sequencing and found to be in complete linkage disequilibrium in a cohort of 96 Leonberger dogs, of which 5 dogs were homozygous and 91 heterozygous for the *GJA9* variant.

**Fig. 2:**
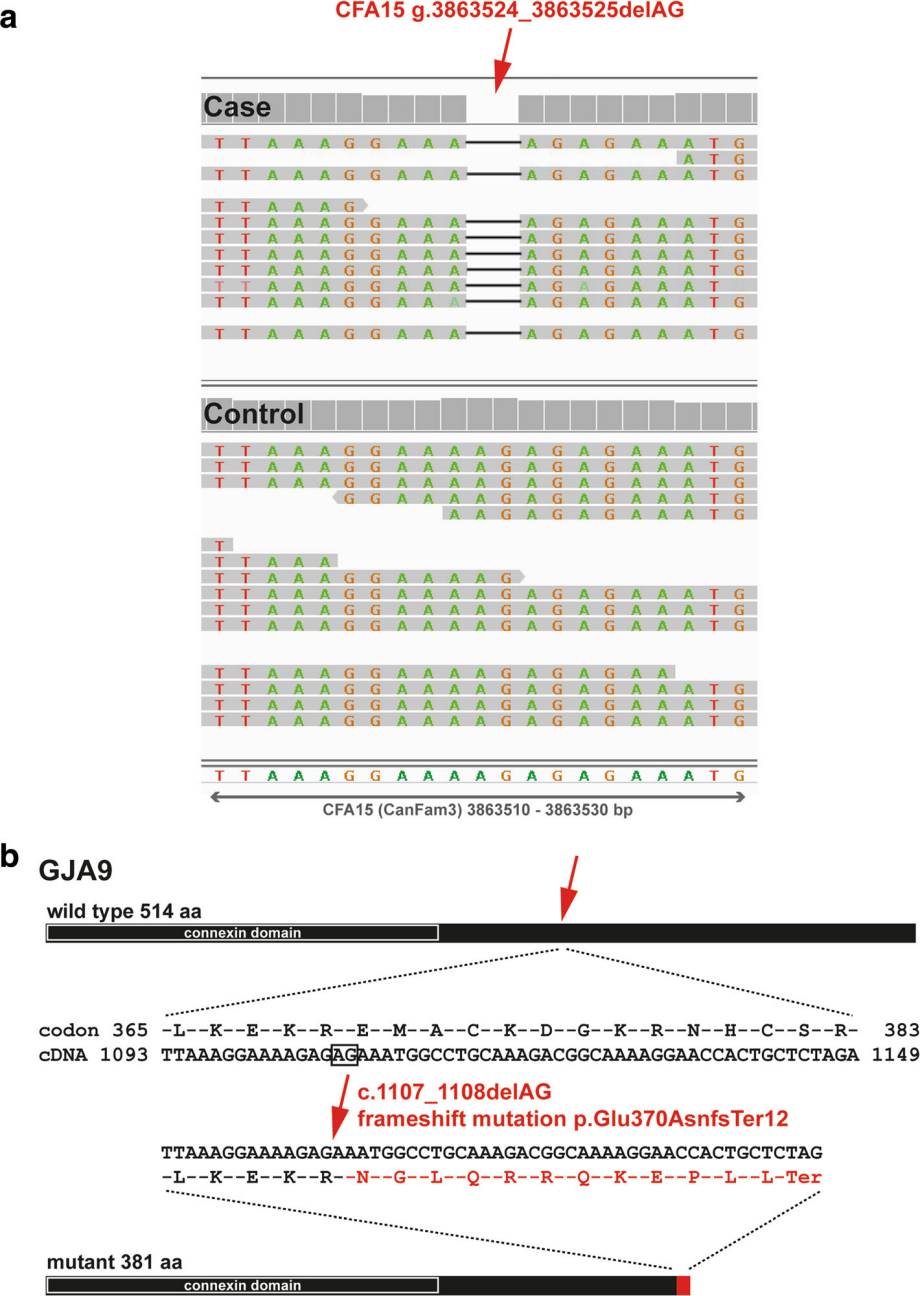
A *GJA9* frameshift variant in polyneuropathy affected Leonberger dogs. **a** IGV screenshots showing the homozygous presence of the 2 bp deletion in the *GJA9* gene on CFA15 in reads from the sequenced case (marked by a red arrow) compared to a control dog genome. **b** The schematic representation indicates that the 2 bp deletion leads to a frameshift that is predicted to produce a significantly truncated intracellular C-terminus of canine GJA9. Note that the transmembranal N-terminal connexin domain that is important for gap junction formation is predicted to be unaffected

The *GJA9* gene was pursued as the most plausible positional candidate gene due to its membership in the connexin gene family known to be causative for human CMT. The identified 2 bp deletion results in a premature stop codon at amino acid residue 382 of the 514 residue full length protein (Fig. 2). Specifically, the *GJA9* variant (CanFam3.1: chr15.3863,524_3863,525delAG) results in a frameshift (ENSCAFT00000038555: c.1107_1108delAG) and premature stop codon (F1PSG8_CANLF: p.Glu370AsnfsTer12) that is predicted to truncate almost half of the intracellular C-terminus of the encoded connexin. Thus we concluded that the *GJA9* deletion is much more likely to cause PN than the other 5 variants.

### LPN2: A dominant inherited form of PN with incomplete penetrance

To investigate the segregation and frequency of the *GJA9* variant, we genotyped in total 7455 Leonberger dogs (Table 1). This includes all 314 Leonberger dogs from the original GWAS cohort, a follow-up cohort of 608 dogs with detailed phenotype records available (Additional file 1), and 6533 mostly young Leonbergers with unknown phenotype status that were submitted for diagnostic purposes, which represent the global population of the breed. There was a highly significant difference in *GJA9* allele frequencies between PN cases and controls from the original GWAS cohort (*p* < 5.5 × 10^−8^), reflected in a difference in genotype frequencies under a dominant model (*p* < 1.2 × 10^−7^), but not a recessive model (*p* < 0.05) (Table 1). These figures give only borderline support for a recessive hypothesis and make it much less well supported than a dominant hypothesis. This result was replicated in the follow-up cohort (genotype frequency different at *p* < 1.9 × 10^−7^, genotype frequency different in a dominant model at *p* < 1.7 × 10^−7^, but not a recessive model at *p* < 0.15) (Table 1). The allele frequency difference in these combined cohorts was significant at *p* < 2.8 × 10^−14^ and the genotype frequency difference under the dominant model was significant at *p* < 6.8 × 10^−14^, clearly indicating dominant inheritance.

**Table 1:** *GJA9* c.1103_1104delAG genotype frequencies in three Leonberger cohorts

Polyneuropathy status	Total	wt/wt (homozygous normal; N/N)	wt/del (heterozygous; D/N)	del/del (homozygous mutant; D/D)
GWAS cohort	314	0.82 (258)	0.16 (51)	0.02 (5)
Affected^a^	176	0.72 (127)	0.25 (44)	0.03 (5)
Non-affected^a,b^	138	0.95 (131)	0.05 (7)	0
Follow-up cohort	608	0.87 (527)	0.13 (79)	0.003 (2)
Affected^a^	296	0.79 (235)	0.20 (59)	0.006 (2)
Non-affected^a,b^	312	0.94 (292)	0.06 (20)	0
Total	7455	0.94 (6995)	0.06 (443)	0.002 (17)
Affected^a^	474	0.76 (362)	0.22 (105)	0.02 (7)
Non-affected^a,b^	450	0.94 (423)	0.06 (27)	0
Unknown^c^	6533	0.95 (6210)	0.05 (313)	0.002 (10)

^a^Only dogs that were homozygous wild type or heterozygous *ARHGEF10* carriers

^b^Only dogs ≥8 years old that showed no signs of PN

^c^Dogs without known phenotype status that were submitted for diagnostic purposes

**Fig. 3:**
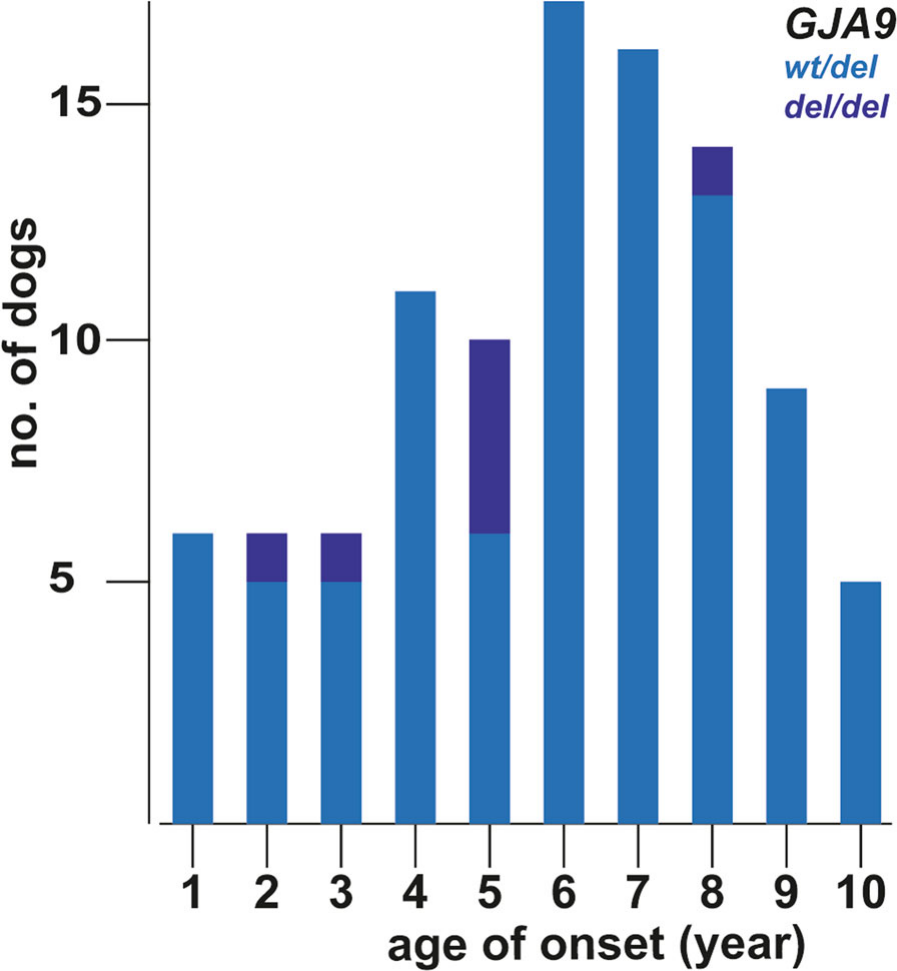
Age of onset of clinical signs in dogs homozygous or heterozygous for the *GJA9* mutation. Age-of-onset of clinical signs for the 100 PN cases in which one or two mutant *GJA9* alleles were present

We then analyzed 137 dogs that carried at least one copy of the *GJA9* mutant allele to determine age of onset and penetrance of the mutant allele (Additional file 1). The owner-reported mean average age of onset in 100 dogs that showed clinical signs was 6 years, and varied from 1 to 10 years (Fig. 3). Due to the limited number of *GJA9* homozygous mutant PN-affected dogs it was not possible to assess whether heterozygous or homozygous PN cases developed clinical signs at different stages in life. Both homozygous mutant and heterozygous dogs of both sexes developed PN, again consistent with an autosomal dominant pattern of inheritance. By 8 years of age 84 of the 137 (61%) dogs carrying the mutant allele showed signs of PN, and within their lifetime 110 of the 137 (80%) showed signs, indicative of a reduced penetrance (Additional file 1). Lastly, the frequency of the *GJA9* variant in the global population of Leonbergers was estimated as 0.032 (Table 1).

A total of 528 Leonbergers in our sample database were diagnosed with PN when the 56 additional PN-affected dogs homozygous for the deletion in *ARHGEF10* were included. Thus, the disease phenotype of approximately 11% (56/528) of all PN-affected Leonbergers could be explained by homozygosity for the *ARHGEF10* (LPN1) variant, whereas homozygosity or heterozygosity for the herein presented PN-associated variant in the *GJA9* gene, which we now term LPN2, explains 21% (110/528) of all cases. Of the remaining 362 PN cases, 85 are LPN1 heterozygous and 277 PN-affected dogs (52%) do not carry either known variant (Additional file 1). Because it is still unclear whether having one copy of the LPN1 allele is sufficient to cause disease, we examined the LPN1 allele frequency in remaining cases (0.12) compared to controls (0.07). This provides evidence of some additional genotypic risk associated with the possession of a single copy of the *ARHGEF10* variant, albeit at a level below 2. Altogether, these allele frequencies continue to present ambiguity (*p* = 6.7 × 10^−4^) in interpreting the correct mode of inheritance of the LPN1 allele (Additional file 1).

### Indistinguishable histopathology in PN affected Leonberger dogs

**Fig. 4:**
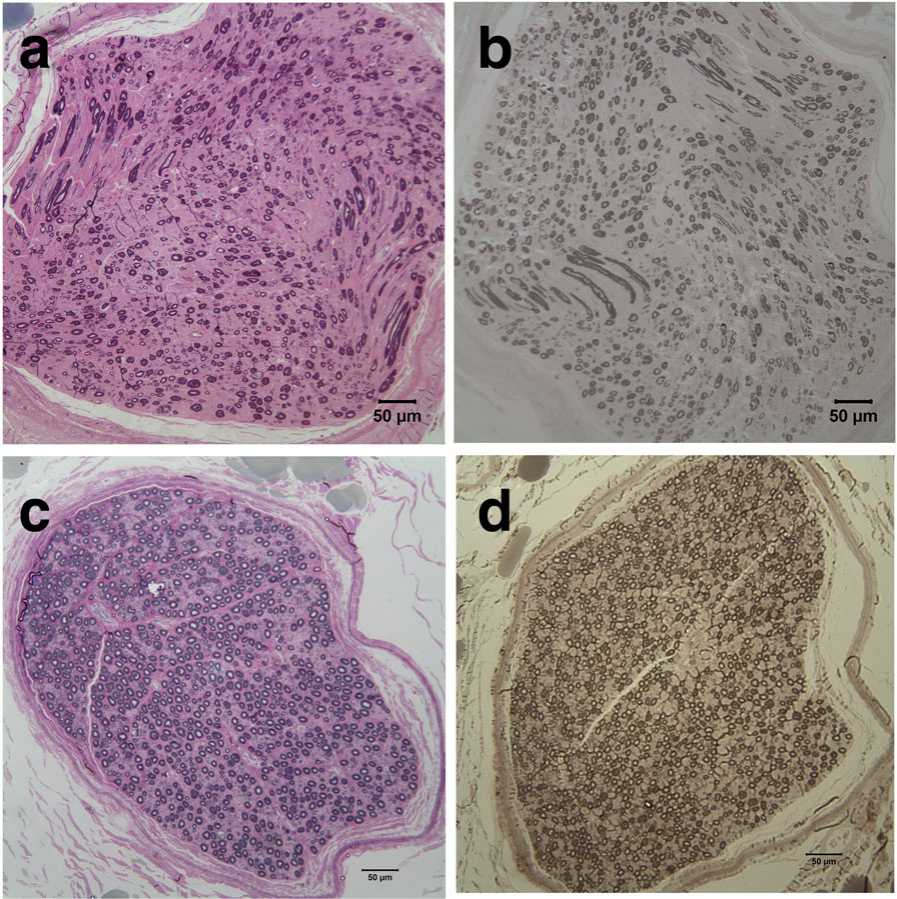
Representative histopathology of the peroneal nerve from male Leonberger dog with polyneuropathy and heterozygous *GJA9* genotype. Resin embedded peroneal nerve sections collected post-mortem from a 5 year old male intact Leonberger dog with clinical signs of polyneuropathy. **a** Toluidine blue-acid fuchsin stain showing loss of predominantly large myelinated fibers and qualitatively increased populations of small calibre nerve fibers. **b** Paraphenelenediamine stain for myelin showing thin but intact myelin sheaths in many small nerve fibers. For comparison, images (**c**, toluidine blue-acid fuchsin stain) and (**d**, paraphenelenediamine stain) are from the peroneal nerve of a 6 year old female neutered Leonberger dog that was negative for both the *ARHGEF10* and *GJA9* variants and without clinical signs of polyneuropathy

Resin sections from the peroneal nerve were qualitatively evaluated from 5 Leonberger dogs with PN and the *GJA9* variant (LPN2 heterozygous) and were compared to Leonberger dogs with PN and the *ARHGEF10* variant (LPN1 homozygous). None of the sections evaluated were from dogs positive for both the *GJA9* and *ARHGEF10* variants. The prominent pathologic abnormality was variably severe nerve fiber loss resulting from chronic axonal degeneration (Fig. 4). Large nerve fiber loss was most prominent with an increased population of small caliber nerve fibers. Similar changes were present in all nerves regardless of the genetic variant.

## Discussion

Inherited canine PN has clinical similarities to the genetically heterogeneous CMT peripheral neuropathies in people. PN in the Leonberger breed shows a variable expression of clinical signs and different ages of onset from <1 year up to 10 years of age. The previously identified *ARHGEF10* frame shift deletion (termed LPN1) causes a severe early-onset disease [11]. Initially, the LPN1 variant explained over 20% of cases; however, due to widespread adoption of genetic testing by breeders, we have not observed a LPN1 case born since 2011. Consequently, the proportion of PN cases explained by the LPN1 mutation has dropped precipitously; currently it explains only approximately 11% of PN cases. Here, our search to identify additional PN genes employed a GWAS with 176 PN affected Leonbergers, which were not homozygous carriers of the recessive *ARHGEF10* loss-of-function variant, and 138 controls. Our present finding of the *GJA9* variant strongly suggests a causative allele for a second inherited form of PN (LPN2), with a wider range of ages of onset and clinical severity, which explains an additional 21% of PN in this breed. These results further confirm genetic similarities between Mendelian forms of PN in dogs and humans. The characteristic axonal degeneration and nerve fiber loss affecting long, large diameter nerves in PN-affected Leonberger dogs resembled mixed forms of human CMT [5]. While qualitative evaluation of peripheral nerve histopathology can confirm the presence of chronic axonal degeneration, nerves can only respond in a limited number of ways to insult of any type and may not be of value in distinguishing LPN variants in Leonberger dogs. Quantitative studies are in progress. The identification of the *ARHGEF10* variant finally confirmed the previously assumed similar pathogenesis of PN in Leonberger dogs to CMT disease in people.

The herein identified 7.7 Mb-sized disease-associated haplotype contained a total of 98 private variants. All 6 private coding variants were experimentally confirmed to occur in complete linkage disequilibrium, the additional 92 non-coding variants were not analyzed further, because the frameshift variant in *GJA9* affects a putative candidate gene, and the variants in *MYCL* and *MACF1* can most likely be ruled out by non-conservation. *GJA9* encodes connexin 59, a connexin gap junction family protein, whose members have emerged as important components of peripheral myelinated nerve fibers. A total of 21 human connexin genes are identified (20 in the mouse), which can be classified into 5 groups based on sequence homology [16]. Six connexin subunits oligomerize to form a hemichannel (also known as connexon) and hemichannels from two adjacent cells dock together at their extracellular domains to form a functional gap junction channel that mediates direct intercellular communication in many physiological processes [16]. Cells and tissues commonly express more than one type of connexin, enabling the formation of homomeric and heteromeric gap junctions [17], and junctional communication between different cells often requires such heterotypic gap junctions [16].

Diverse genetic diseases in humans, including disorders of the nervous system, are caused by variants in connexin genes [18]. The *GJB1* gene, with more than 400 known variants, is causally implicated in one of the five most common CMT subtypes in people [OMIM 304040; 19,20]. The encoded connexin 32 forms gap junctions between the folds of Schwann cell membranes. These Schwann cells wrap around the axons of peripheral nerves and form a layer of myelin that is critical for the conduction of nerve impulses. Mutations in *GJB1* appear to hinder diffusion across the concentric layers of myelin, resulting in disruption of myelin and hence axonal degeneration [19, 20]. Due to the male sex bias, similar phenotype, and the slowly progressive nature of the PN disease in Leonberger dogs, it was initially speculated, that variants in the canine ortholog of *GJB1* encoding connexin 32 could be responsible [21]. Our identification of a variant in the related gap junction protein GJA9 nicely confirms these earlier assumptions. Interestingly, as we have observed in our canine study, there is a high phenotypic variability within CMT genotypes and variant-specific manifestations between different human CMT types [22]. Recently, the human *GJA9* gene, among others, was reported as novel candidate disease gene for neurogenetic disorders after the first results from clinical exome sequencing of 149 patients with various neurocognitive phenotypes [23]. But, to our knowledge, there is as yet no non-synonymous *GJA9* variant known to be associated with CMT in people. Therefore this canine *GJA9* associated form of PN provides an interesting animal model and adds this gene to the list of candidates for human CMT. This is especially of value as there is no *GJA9* ortholog in the mouse genome [24]. Finally, in the light of the current status of the dog reference genome and its incomplete annotation, we acknowledge that the list of private variants of the disease-associated haplotype is most likely not complete. In addition to the fact that we were not able to exclude the reported 97 private variants other than the *GJA9* frameshift variant, one could assume that other undetected variants located in existing reference sequence gaps or unrecognized structural variants exist on the disease-associated haplotype.

The age-of-onset analysis indicates that LPN2 is a dominantly inherited trait with incomplete penetrance. Several heterozygous dogs >8 years of age still have not shown clinical signs of weakness. This might suggest that nerve cells can still function reasonably well with a single copy of the normal allele, or even the absence of the GJA9 protein entirely. The predicted protein from the mutant allele contains <75% of the amino acids present in the wild type protein. It is possible that since the four N-terminal transmembrane domains forming the connexin channel domain are unchanged, the identified variant with its significantly shortened intracellular C-terminus may not be a complete loss-of-function allele. Other mechanisms to explain the somewhat complex genotype-age of onset-phenotype relationship include the possibilities that other connexins partially serve the function of GJA9, a dominant-negative effect of the mutant protein, or simple haploinsufficiency affected by genetic background. We further suggest that the incomplete association between *GJA9* genotype and PN in our large population is partly due to contributions from phenocopies, misdiagnoses, as well as genetic heterogeneity.

These findings have serious implications for breeders, as genetically susceptible dogs may not develop clinical disease until later in life, if at all, and often well after a dog may have bred multiple times. Since the offering of the LPN2 test to the Leonberger community nearly 7500 dogs have been genotyped for the *GJA9* deletion. Although this is not a random sample of the breed, the finding of a mutant allele frequency of approximately 0.03, and its dominant nature, indicates it should be considered a serious problem for the health and welfare of breed.

## Conclusions

Highly probable causative variants in the *ARHGEF10* and *GJA9* genes have now been identified for two forms of PN in Leonberger dogs. PN in this breed exhibits remarkable variation in phenotypic severity and age of onset among affected dogs, suggesting the influence of modifiers of both the *GJA9* form and additional forms not explained by either known variant. It is likely that many more Leonberger dogs with PN will be needed to improve the power to detect loci that contribute to the non-*ARHGEF10* /*GJA9* form(s) of PN in Leonbergers, which might represent a multigenic complex trait. Characterization of genomic variation underlying PN phenotypic variation in this breed with small effective population size will present a powerful resource for understanding the molecular causes behind variable canine polyneuropathy phenotypes. Finally, this study adds *GJA9* for the first time to the list of candidate genes for human CMT.

## Additional files

Additional file 1: Phenotype records and GJA9 genotypes of 922 Leonberger dogs with detailed phenotype records. (XLSX 469 kb)

Additional file 2: GWAS results comparing 176 cases vs. 138 controls. (XLSX 11371 kb)

Additional file 3: Haplotype reconstruction for the centromeric region of acrocentric CFA15 of 314 dogs. (XLSX 3145 kb)

Additional file 4: List of 98 private sequence variants of the sequenced PN-affected Leonberger in the critical region on CFA15. (XLSX 31 kb)

## Abbreviations

BAM: Binary version of a sequence alignment/map (SAM) file
bp: Base pairs
CMT: Charcot-Marie-Tooth
GJA9: Gap junction protein alpha 9
GWAS: Genome wide association study
IGV: Integrative Genomics Viewer
LPN1: Leonberger polyneuropathy type 1
LPN2: Leonberger polyneuropathy type 2
OMIA: Online Mendelian Inheritance in Animals
PCR: Polymerase chain reaction
SNP: Single nucleotide polymorphism
